# Refinement of microbiota analysis of specimens from patients with respiratory infections using next-generation sequencing

**DOI:** 10.1038/s41598-021-98985-8

**Published:** 2021-10-01

**Authors:** Hiroaki Ikegami, Shingo Noguchi, Kazumasa Fukuda, Kentaro Akata, Kei Yamasaki, Toshinori Kawanami, Hiroshi Mukae, Kazuhiro Yatera

**Affiliations:** 1 Department of Respiratory Medicine, University of Occupational and Environmental Health, Japan, 1-1 Iseigaoka, Yahatanishi-ku, Kitakyushu-city, Fukuoka, 807-8555 Japan; 2 Department of Microbiology, University of Occupational and Environmental Health, Japan, Kitakyushu, Japan; 3 Department of Respiratory Medicine, Nagasaki University Graduate School of Biomedical Sciences, Nagasaki, Japan

**Keywords:** Bacterial infection, Infectious diseases, Respiratory tract diseases

## Abstract

Next-generation sequencing (NGS) technologies have been applied in bacterial flora analysis. However, there is no standardized protocol, and the optimal clustering threshold for estimating bacterial species in respiratory infection specimens is unknown. This study was conducted to investigate the optimal threshold for clustering 16S ribosomal RNA gene sequences into operational taxonomic units (OTUs) by comparing the results of NGS technology with those of the Sanger method, which has a higher accuracy of sequence per single read than NGS technology. This study included 45 patients with pneumonia with aspiration risks and 35 patients with lung abscess. Compared to Sanger method, the concordance rates of NGS technology (clustered at 100%, 99%, and 97% homology) with the predominant phylotype were 78.8%, 71.3%, and 65.0%, respectively. With respect to the specimens dominated by the *Streptococcus mitis* group, containing several important causative agents of pneumonia, Bray Curtis dissimilarity revealed that the OTUs obtained at 100% clustering threshold (versus those obtained at 99% and 97% thresholds; medians of 0.35, 0.69, and 0.71, respectively) were more similar to those obtained by the Sanger method, with statistical significance (*p* < 0.05). Clustering with 100% sequence identity is necessary when analyzing the microbiota of respiratory infections using NGS technology.

## Introduction

Bacterial pneumonia is a major infectious disease worldwide, and the number of patients with pneumonia is predicted to increase, particularly in Japan, where the population is rapidly aging. Therefore, it is important to properly evaluate the bacteriological etiology to provide optimal treatments for these patients in clinical practice^1^. Next-generation sequencing (NGS) technologies have been applied in bacterial flora analysis, and advances in these methods over the last decade have revealed the presence of microbiota in the lower respiratory tract, which was previously considered as sterile^2–4^. The use of NGS technology has become more common in microbiota analysis, including to assess respiratory infections^5–7^, but there are some limitations in interpreting the results.

In NGS analysis, technical methods, such as those used for the selection of target genes and gene region or length, as well as equipment are diverse. For example, untargeted metagenomic NGS of clinical samples may be the most promising approach for comprehensively detecting agents responsible for infectious diseases and evaluating normal flora, whereas the analysis of large datasets requires a combination of bioinformatics skills; therefore, few laboratories can use NGS diagnostically^8,9^. Targeted sequencing, including sequencing part of the 16S ribosomal RNA (rRNA) gene, is a superior approach for detecting and estimating bacterial species compared to metagenomic NGS methods because of their low complexity, low cost, and practical clinical application. However, the analysis pipelines, including assemblers and analytical parameters, can significantly influence the final results, and incorrect sequence results have been reported^10,11^. There is no standardization between amplicon studies with NGS technology^12^, leading to issues with research replication^13^. Clustering of 16S rRNA sequences into operational taxonomic units (OTUs) was generally analyzed in the gastrointestinal and urological areas with thresholds of 99% and 97%, respectively^14–17^, but it is unclear whether this approach is applicable to the microbiota in respiratory specimens. In recent years, analysis based on amplicon sequence variants (ASVs) has proven to be useful because it increases the accuracy of analysis per sequence compared to OTU analysis^18–20^. However, several problems have been highlighted regarding ASV analysis^21–23^, such as the possibility that species of the genus *Streptococcus* represented by *Streptococcus pneumoniae*, which are important in respiratory infections, are underestimated^21^. Therefore, further validation of the method is required before use in the assessment of pneumonia with aspiration risks in which *Streptococcus* species are frequently detected. OTU analysis has been utilized in various fields of research^14,24,25^, and verification of the accuracy of this method is required.

A disadvantage of performing clone library analysis using the Sanger method is the reduced analysis capacity per operation compared to NGS technology; however, the Sanger method can read comparatively long sequences (700–1000 bp) with high accuracy (99.999%)^26^ relative to NGS and can estimate the species level for some bacteria^27^. The combination of the clone library and Sanger methods can be used to estimate predominant bacterial species without the use of culture-based methods in clinical specimens. We have reported the usefulness and significance of this approach in evaluating respiratory infections^28–33^.

The genus *Streptococcus*, including *Streptococcus pneumoniae* or *Streptococcus intermedius*, is a major causative agent of bacterial pneumonia in elderly people^30,34^ and in patients with lung abscess^31^. *Streptococcus pneumoniae* is generally the major causative agent in respiratory infections, but species such as, oral streptococci, *Streptococcus pseudopneumoniae*^35,36^, and *S. intermedius*^37,38^ are important in estimating antibiotic resistance and the clinical course of the disease. The nucleotide sequences of 16S rRNA regions of these organisms, including *S. pneumoniae* and *Streptococcus mitis*, are very similar^27^, and thus correct nucleotide sequence identification is crucial; however, it is unclear whether these bacteria can be distinguished by targeted NGS analysis.

Respiratory infections exhibit diverse bacterial flora even in each sample directly obtained from the lung, which are difficult to characterize using commercially available bacterial cultures. Therefore, we retrospectively evaluated bronchoalveolar lavage fluid (BALF) samples from patients with pneumonia with aspiration risks and from patients with lung abscess, in which the genus *Streptococcus* plays an important role, to compare the results of the Sanger method combined with the clone library method and NGS methods. We also investigated the optimal threshold for clustering 16S rRNA gene sequences into OTUs by NGS technology by comparing the results of NGS technology with those of the Sanger method.

## Methods

### Study population

**Figure 1:**
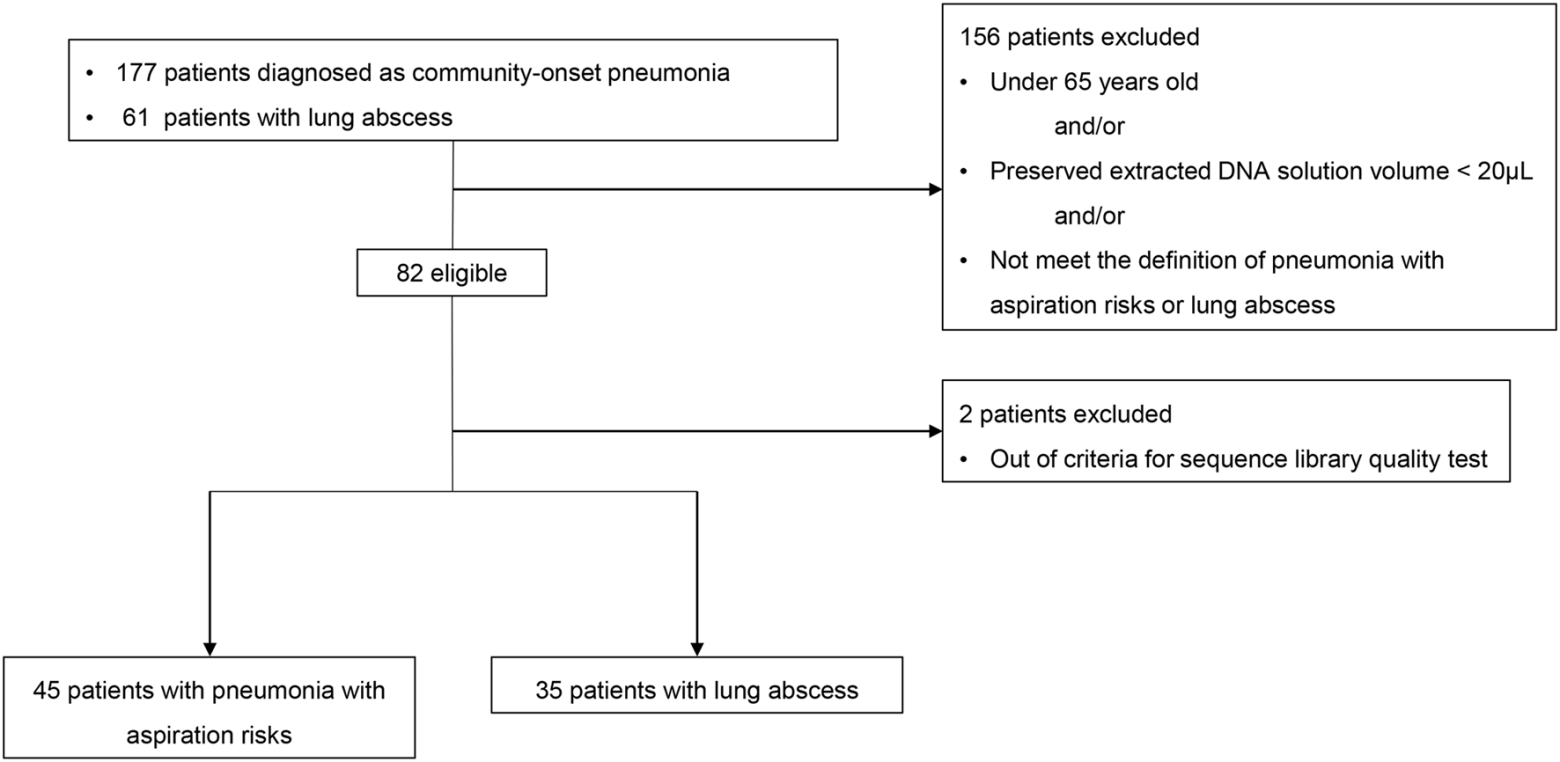
Patient inclusion and exclusion flow diagram.

This was a retrospective study targeting 177 patients with community-onset pneumonia (community-acquired pneumonia and healthcare-associated pneumonia) and 61 patients with lung abscess on whom a bronchoscopy examination was performed at the University of Occupational and Environmental Health, Japan, and affiliated community hospitals between April 2010 and March 2016. Most patients were included in previous studies^30,31^. Patients less than 65 years of age and those who did not meet the definition of pneumonia with aspiration risks and lung abscess were excluded^30,31^. Two cases were also excluded during quality verification of the BALF samples. Finally, 45 of 177 patients with pneumonia with aspiration risks and 35 of 61 patients with lung abscess were included in this study (Fig. 1). This study was approved by the Ethics Committee of Medical Research, University of Occupational and Environmental Health, Japan (UOEHCRB18-016). All experiments were performed in accordance with relevant guidelines and regulations. The need for written informed consent was waived by the Ethics Committee of Medical Research, University of Occupational and Environmental Health, Japan because of the retrospective study design.

### Definitions

Community-onset pneumonia was defined according to the guidelines of the Infectious Diseases Society of America/American Thoracic Society^39^. Aspiration risks were defined according to the criteria of Marik et al.^40^ as in our previous reports^30,31^ and included neurologic dysphagia, disruption of the gastroesophageal junction, or anatomical abnormalities of the upper aerodigestive tract.

Lung abscess was diagnosed when the following three criteria were fulfilled: (a) the presence of new areas of infiltrations with a cavity or low-density area within the infiltrates on chest radiographs and/or computed tomography; (b) new clinical findings including at least two of the following: fever, sputum production, coughing, chest pain, and leukocytosis (peripheral white blood cell count ≥ 10,000/μL), and (c) exclusion of other causes^31^.

### Sample collection

Fiberoptic bronchoscopy was performed as previously described^29^. Briefly, BALF samples were obtained using 40 mL of sterile saline from the affected lesions of patients with pneumonia or lung abscess.

### Cell lysis efficiency analysis and DNA extraction

DNA samples were extracted from the BALF samples by adding sodium dodecyl sulfate (final concentration: 3.0%) and glass beads followed by vigorous shaking, as previously described^29^.

### Microbiota analysis using the Sanger method

The partial 16S rRNA gene fragments (approximately 550 bp) were amplified by polymerase chain reaction (PCR) with universal primers (E341F and E907R), as previously described^29^. The amplified products were cloned into *Escherichia coli* using a TOPO TA cloning kit (Invitrogen, Carlsbad, CA, USA). Nucleic acid sequences of 96 randomly selected clones in each clone library were determined using a 3130*xl* Genetic Analyzer (Applied Biosystems, Foster City, CA, USA). Highly accurate sequences selected by Phred quality values were trimmed from the primer and vector regions, and only sequences with good quality were used for analysis^29^.

### NGS library preparation and Illumina MiSeq sequencing

Extracted DNA samples were purified using Agencourt AMPure XP (Beckman Coulter, Brea, CA, USA). Two-step PCRs were performed on the purified DNA samples to obtain sequence libraries. The first PCR was an amplification step performed using a 16S (V3‒V4) Metagenomic Library Construction Kit for NGS (Takara Bio, Shiga, Japan) with primer pairs, 341F (5′-TCGTCGGCAGCGTCAGATGTGTATAAGAGACAGCCTACGGGNGGCWGCAG-3′) and 806R (5′-GTCTCGTGGGCTCGGAGATGTGTATAAGAGACAGGGACTACHVGGGTWTCTAAT-3′), corresponding to the V3‒V4 region of the 16S rRNA gene. Thermal cycling was performed in a TaKaRa PCR Thermal Cycler Dice Gradient under the following conditions: initial denaturation at 94 °C for 1 min, followed by 28 cycles of denaturation at 98 °C for 10 s, annealing at 50 °C for 15 s, and extension at 68 °C for 15 s. PCR amplicons were purified using AMPure XP magnetic purification beads.

The second PCR was performed to add the index sequences for Illumina sequencing using a Nextera XT Index kit v2 (Illumina, San Diego, CA, USA) and Tks Gflex DNA polymerase S (Takara Bio). Thermal cycling was performed in a TaKaRa PCR Thermal Cycler Dice Gradient (Takara Bio) with the following conditions: initial denaturation at 94 °C for 1 min, followed by 8 cycles of denaturation at 98 °C for 10 s, annealing at 60 °C for 15 s, and extension at 68 °C for 15 s. PCR amplicons were evaluated for quality verification of the sequence library using an Agilent 4200 TapeStation (Agilent Technologies, Santa Clara, CA, USA).

After quality verification, mixed samples were prepared by pooling approximately equal amounts of each amplified DNA and sequenced using MiSeq Reagent Kit V3 (250 × 2 cycles) and a MiSeq sequencer (Illumina), according to the manufacturer’s instructions. The obtained nucleotide sequences were subjected to assembly and clustering processes using the CD-HIT-OTU algorithm^41^. First, low-quality reads (reads containing > 10 bases with an error rate ≥ 10%, more than 4 mismatches in the overlap region, or non-overlapping paired reads) were removed and assembled. The assembled sequences with 100% homology were clustered, and chimera sequences were removed. In addition, clusters with a low frequency (39 contigs or less) of occurrence estimated using simulations from the maximum cluster size were removed. The nucleotide sequences obtained were clustered based on homologies of 100%, 99%, and 97%. A representative sequence was selected; those of OTUs showed the longest lengths.

### Homology searching

In the Sanger method, the resulting high-accuracy reads were directly homology searched, whereas for NGS technology, homology searching was performed for representative sequences of each OTU. The determined sequences were compared with an in-house database containing the 16S rRNA gene sequences of 5,878 type strains using the basic local alignment search tool (BLAST; NCBI, Bethesda, MD, USA) algorithm^42^. Bacterial type strains that were top hits with ≥ 97% homology identified using BLAST were classified as presumptive species^43^, and those with < 97% homology were classified as unidentified organisms^29–31,44^. The 16S rRNA gene sequences of type strains were obtained from the DNA Data Bank of Japan (http://www.ddbj.nig.ac.jp/) and the Ribosomal Database Project (http://rdp.cme.msu.edu/).

### Interpretation of the results

The bacterial phylotype exhibiting the highest proportion of the total microbiota in each BALF sample was defined as the “predominant phylotype.” In addition, phylotypes that occupied less than 5% of each microbiota were classified as “Others,” as previously described^29^.

### Bray Curtis dissimilarity

The Bray Curtis dissimilarity was calculated for each specimen to assess the degree of difference between the results of the Sanger method and NGS technology. This analysis is a standardized index that has a value of 0 when the species composition is similar between two microbiota and a maximum value of 1 when they are completely different^45^. Species included in “Others” and bacteria considered as unclassified organisms were excluded, and major populations were compared.

### Statistical analysis

The statistical software PRISM 8 (GraphPad Software, Inc., San Diego, CA, USA) was used for the Kruskal–Wallis test and post hoc Dunn's test with Bonferroni adjustment for multiple comparisons, as appropriate.

## Results

### Patient characteristics

The characteristics of 80 patients (45 with pneumonia with aspiration risks and 35 with lung abscess) are shown in Table 1. The median age of the patients was 73 [interquartile range (IQR) 70–79] years and the inpatient mortality rate was 6.3%. In many cases (68 of 80 patients), the severity grade according to the Pneumonia Severity Index score^46^ was mild to moderate.

**Table 1:** Characteristics of total patients (n = 80).

	Total (n = 80)	
Age, median (IQR), years	73	(70–79)
Male; n (%)	61	(76.3)
**Smoking status; n (%)**		
Current smoker	11	(13.8)
Ex-smoker	36	(45.0)
**Comorbidity; n (%)**		
Malignancy	17	(21.3)
Cerebrovascular disease	19	(23.8)
Chronic cardiac disease	13	(16.3)
Chronic respiratory disease	20	(25.0)
Chronic liver disease	11	(13.8)
Chronic kidney disease	4	(5.0)
Diabetes mellitus	16	(20.0)
Collagen disease	6	(7.5)
No comorbidity disease	8	(10)
**PSI score; n (%)**		
Mild (I–III)	34	(42.5)
Moderate (IV)	34	(42.5)
Severe (V)	12	(15.0)
In-hospital mortality; n (%)	5	(6.3)

*IQR* interquartile range; *PSI score* Pneumonia Severity Index score.

### Comparison of the results of microbiota analysis between the Sanger method and NGS

There were 6,530 total clones, and the mean nucleotide sequence length using the Sanger method was 544.3 bases (per read). NGS generated 7,637,916 pairs of reads, and the total number of contigs after filtering and assembling was 4,103,775 with a mean nucleotide sequence length of 459.5 (see Supplementary Table S1 online). OTUs were created with 100%, 99%, and 97% clustering thresholds for subsequent analysis. The total numbers of OTUs clustered at 100%, 99%, and 97% identity were 3,663, 493, and 255, respectively. The rarefaction curves of the number of OTUs identified per sample almost plateaued with ~ 5000 sequence reads (see Supplementary Fig. S1 online).

**Figure 2:**
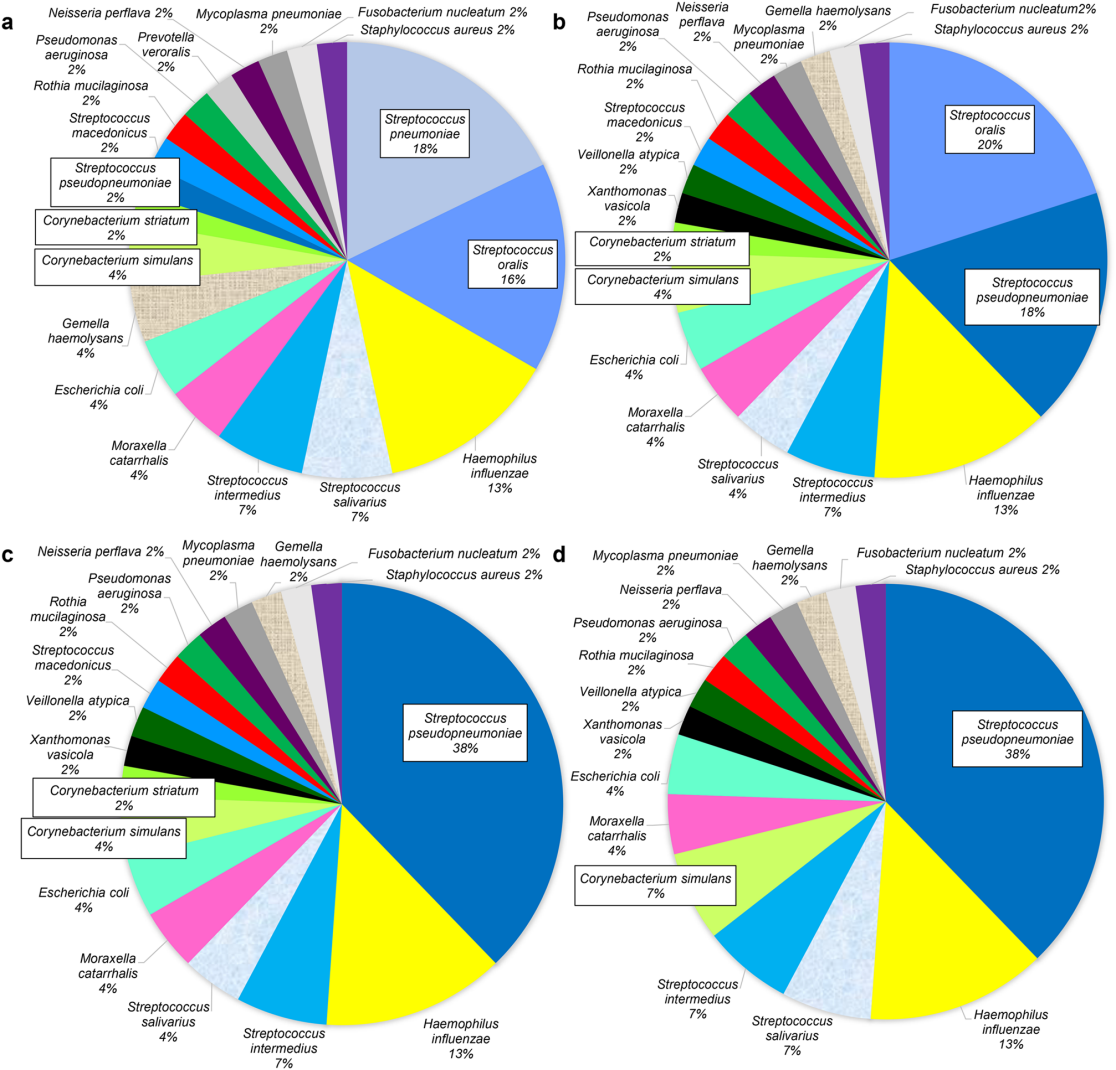
Predominant phylotype by the Sanger method and NGS technology (pneumonia with aspiration risks). This pie chart shows the percentage of predominant bacterial phylotypes in the bronchoalveolar lavage fluid for the Sanger method (**a**), NGS (100%) (**b**), NGS (99%) (**c**), and NGS (97%) (**d**) for patients with pneumonia with aspiration risks. The same color represents the same bacterial phylotypes. NGS (100%) = NGS results obtained with a clustering threshold of 100%; NGS (99%) = NGS results obtained with a clustering threshold of 99%; NGS (97%) = NGS results obtained with a clustering threshold of 97%.

In patients with pneumonia with aspiration risks, the most frequently detected bacterial phylotype by the Sanger method was *S. pneumoniae* (17.8%), followed by *Streptococcus oralis* (15.6%) and *Haemophilus influenzae* (13.3%) (Fig. 2). In contrast, the most frequently detected bacterial phylotype was *S. oralis* (20.0%), followed by *S. pseudopneumoniae* (17.8%), and *H. influenzae* (13.3%) by NGS technology (100% identity thresholds for clustering). *Streptococcus pseudopneumoniae* (37.8%) was the most abundant phylotype, followed by *H. influenzae* (13.3%) in NGS technology (99% and 97% identity thresholds for clustering).

**Figure 3:**
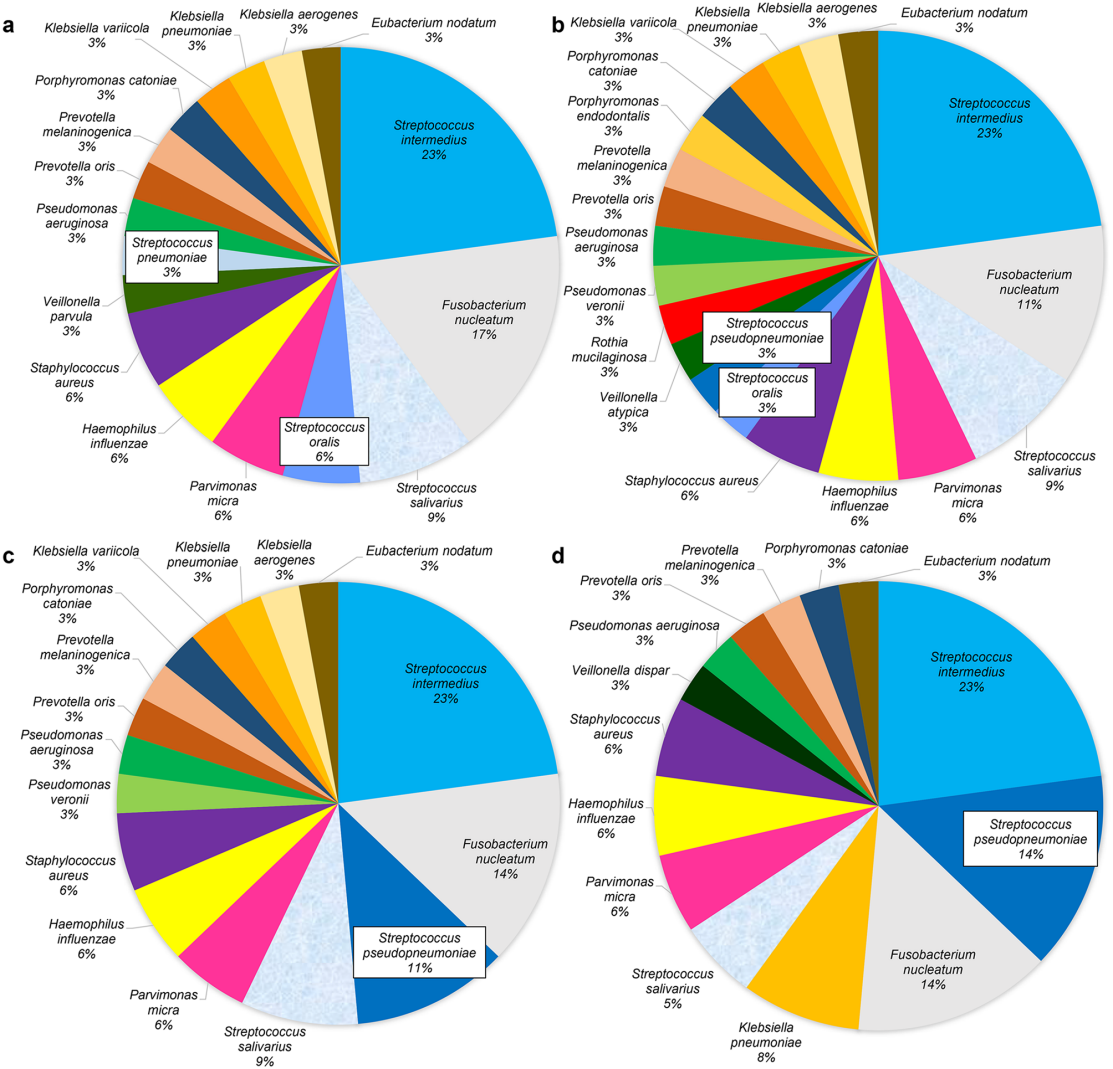
Predominant phylotype by the Sanger method and NGS technology (lung abscess). This pie chart shows the percentage of predominant bacterial phylotypes in the bronchoalveolar lavage fluid for the Sanger method (**a**), NGS (100%) (**b**), NGS (99%) (**c**), and NGS (97%) (**d**) for patients with lung abscess. The same color represents the same bacterial phylotypes. NGS (100%) = NGS results obtained with a clustering threshold of 100%; NGS (99%) = NGS results obtained with a clustering threshold of 99%; NGS (97%) = NGS results obtained with a clustering threshold of 97%.

In lung abscess, the most frequently detected bacterial phylotype by the Sanger method was *S. intermedius* (22.9%), followed by *Fusobacterium nucleatum* (17.1%) and *Streptococcus salivarius* (8.6%) (Fig. 3). Alternately, the most frequently detected bacterial phylotype was *S. intermedius* (22.9%), followed by *F. nucleatum* (11.4%) and *S. salivarius* (8.6%) in NGS technology (100% identity thresholds for clustering). At 99% and 97% identity thresholds for clustering in NGS technology, *S. intermedius* (22.9%) was the most frequently detected phylotype, followed by *F. nucleatum* (14.3%) *and S. pseudopneumoniae* (11.4% at 99% identity thresholds for clustering, 14.3% at 97% identity thresholds for clustering).

The concordance rates in the results of the predominant phylotype determined by NGS technology, using 100%, 99%, and 97% identity thresholds for clustering, with those of the Sanger method were 78.8% (63 of 80), 71.3% (57 of 80), and 65.0% (52 of 80), respectively (see Supplementary Table S2 online).

### Bray Curtis dissimilarity of Sanger method versus NGS technology with NGS clustering threshold changes

**Figure 4:**
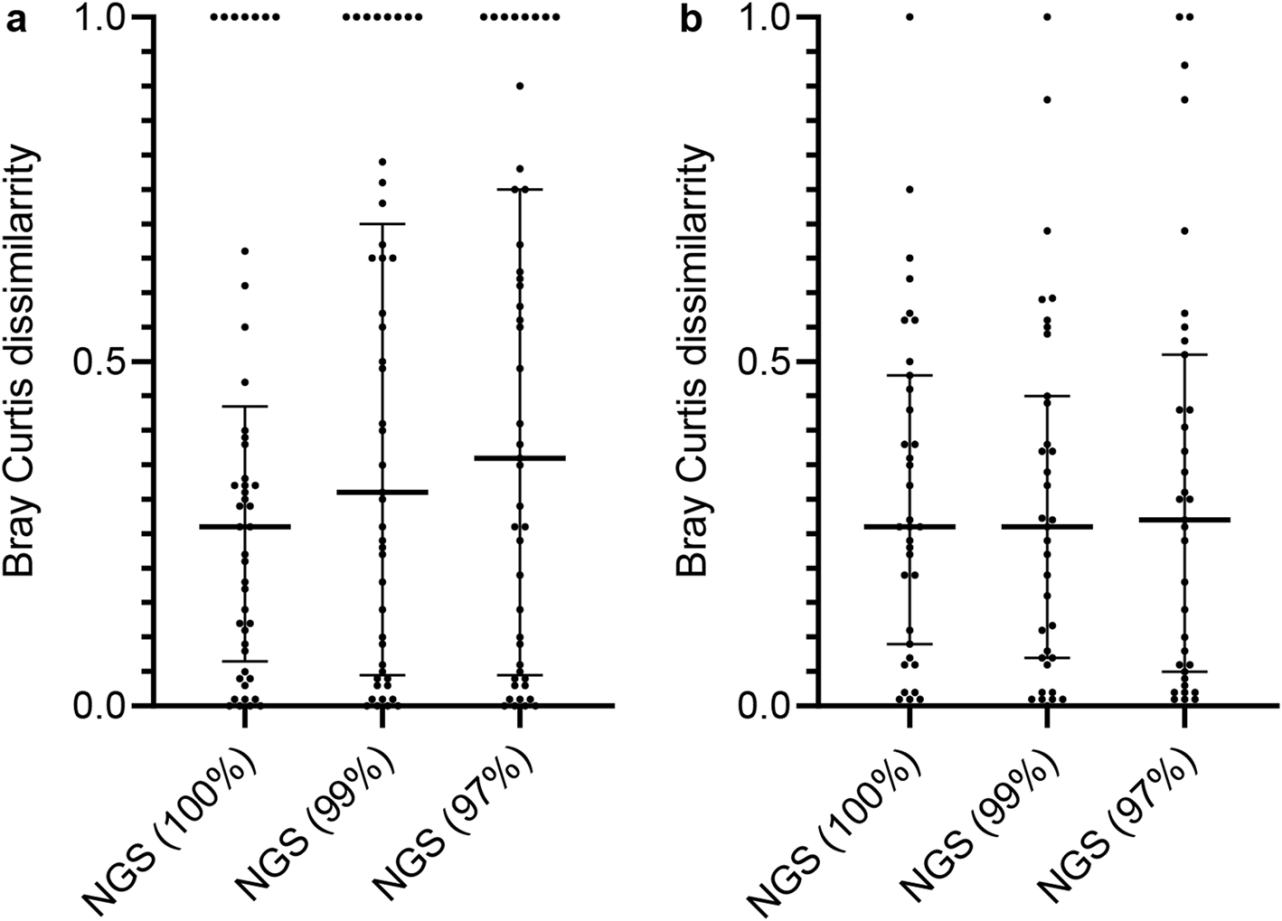
Bray Curtis dissimilarity between the Sanger method and NGS technology. The results of the Bray Curtis dissimilarity to assess the similarity of microbiota analysis between the Sanger method and NGS technology in patients with pneumonia with aspiration risks (**a**) and patients with lung abscess (**b**) showed no difference in flora similarity between clustering thresholds (pneumonia with aspiration risks: *p* = 0.59, lung abscess: *p* = 0.88). *P*-values were determined by Kruskal–Wallis test. NGS (100%) = NGS results obtained with a clustering threshold of 100%; NGS (99%) = NGS results obtained with a clustering threshold of 99%; NGS (97%) = NGS results obtained with a clustering threshold of 97%.

The results of Bray Curtis dissimilarity between the Sanger method and NGS technology showed no differences in microbiota similarity among the 100%, 99%, and 97% clustering thresholds in pneumonia with aspiration risks (NGS (100%): median 0.26 [(IQR) 0.07–0.44] vs NGS (99%): 0.31 [0.05–0.70] vs NGS (97%): 0.36 [0.05–0.75], *p* = 0.59) and lung abscess (NGS (100%): median 0.26 [IQR 0.09–0.48] vs NGS (99%): 0.26 [0.07–0.45] vs NGS (97%): 0.27 [0.05–0.51], *p* = 0.88) (Fig. 4).

**Figure 5:**
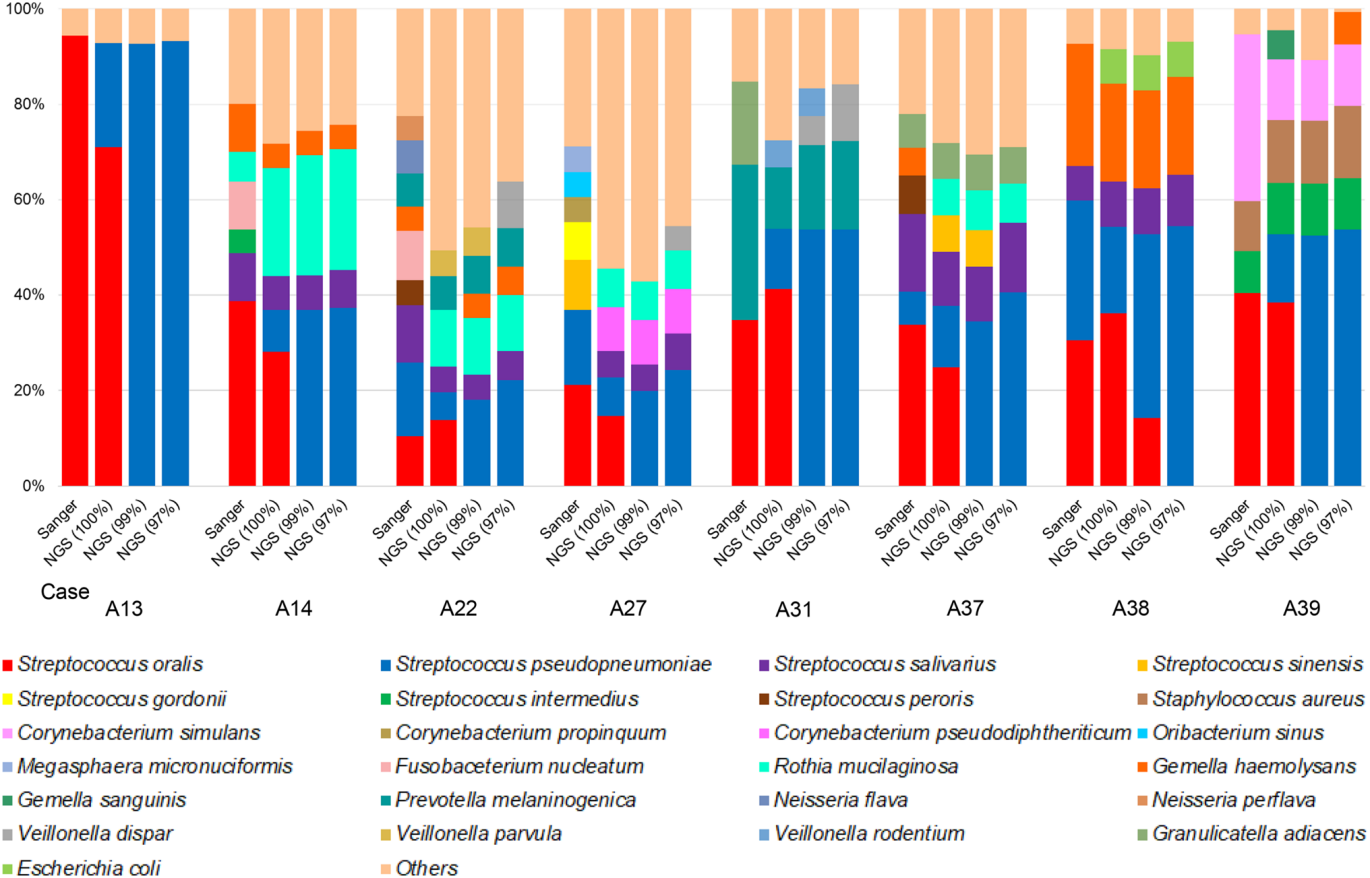
Detected phylotypes using the Sanger method and NGS technology in the eight pneumonia cases in which *Streptococcus mitis* group (other than pneumococcus) was detected predominantly by the Sanger method. NGS (100%) = NGS results obtained with a clustering threshold of 100%; NGS (99%) = NGS results obtained with a clustering threshold of 99%; NGS (97%) = NGS results obtained with a clustering threshold of 97%.

**Figure 6:**
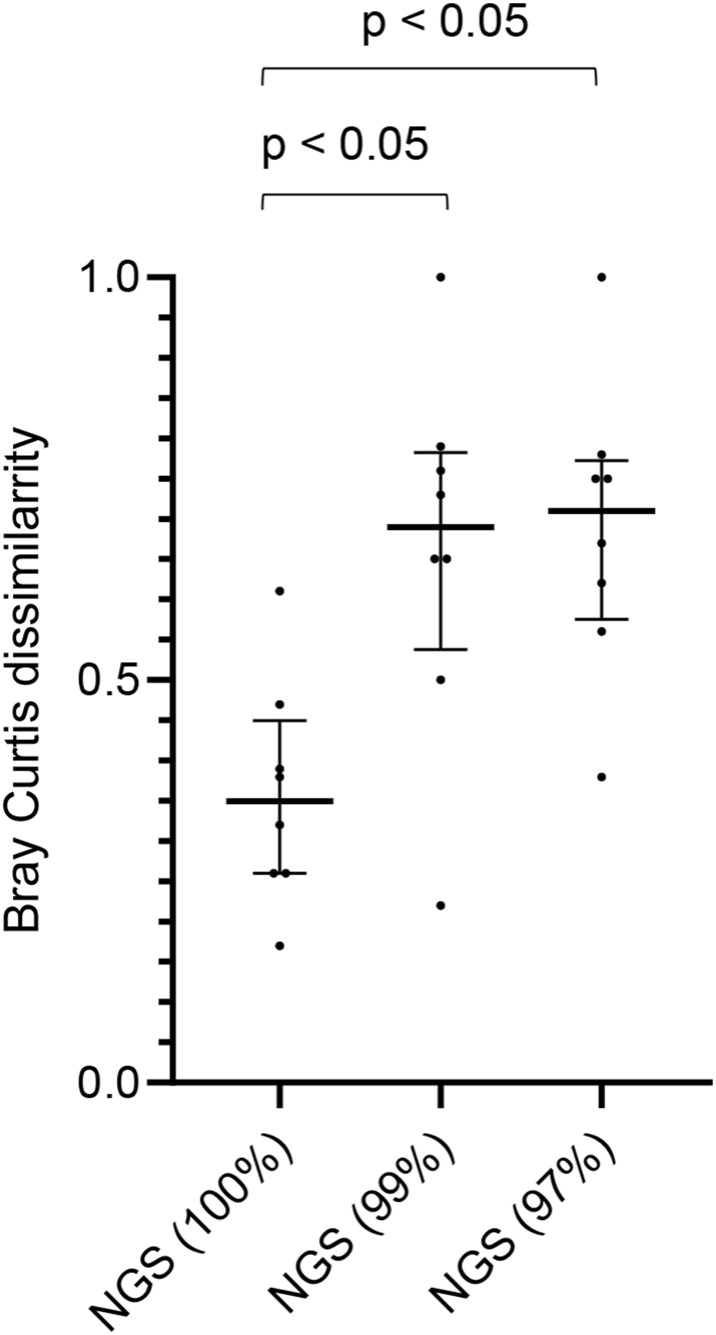
Similarity of the Sanger method and NGS technology using Bray Curtis dissimilarity in the eight pneumonia cases in which *Streptococcus mitis* group (other than pneumococcus) was detected predominantly by the Sanger method. Bray Curtis dissimilarity revealed that OTUs obtained at 100% clustering threshold were more similar to those obtained by the Sanger method than did those obtained at 99% and 97% thresholds (*p* < 0.05). *P*-values were determined by Kruskal–Wallis test and a post hoc Dunn’s test with Bonferroni adjustment for multiple comparisons. NGS (100%) = NGS results obtained with a clustering threshold of 100%; NGS (99%) = NGS results obtained with a clustering threshold of 99%; NGS (97%) = NGS results obtained with a clustering threshold of 97%.

As shown in Fig. 2, the ability to detect bacterial phylotypes in the *S. mitis* group, such as *S. oralis* and *S. pseudopneumoniae*, differed according to the clustering threshold. Based on these results, we compared the results of the Sanger method and NGS technology in eight cases in which the *S. mitis* group (*S. oralis* and *S. pseudopneumoniae*), excluding *S. pneumoniae*, was detected as the predominant bacterial species using the Sanger method in pneumonia with aspiration risks (Fig. 5). Of note, Bray Curtis dissimilarity revealed that the OTUs obtained with the 100% threshold (median 0.35 [IQR 0.26–0.45]) were more similar to those obtained by the Sanger method than to those obtained with the 99% (0.69 [0.54–0.78]) and 97% (0.71 [0.58–0.77]) thresholds (*p* < 0.05) (Fig. 6).

### Total number of OTUs detected as unidentified organisms in each sample

**Figure 7:**
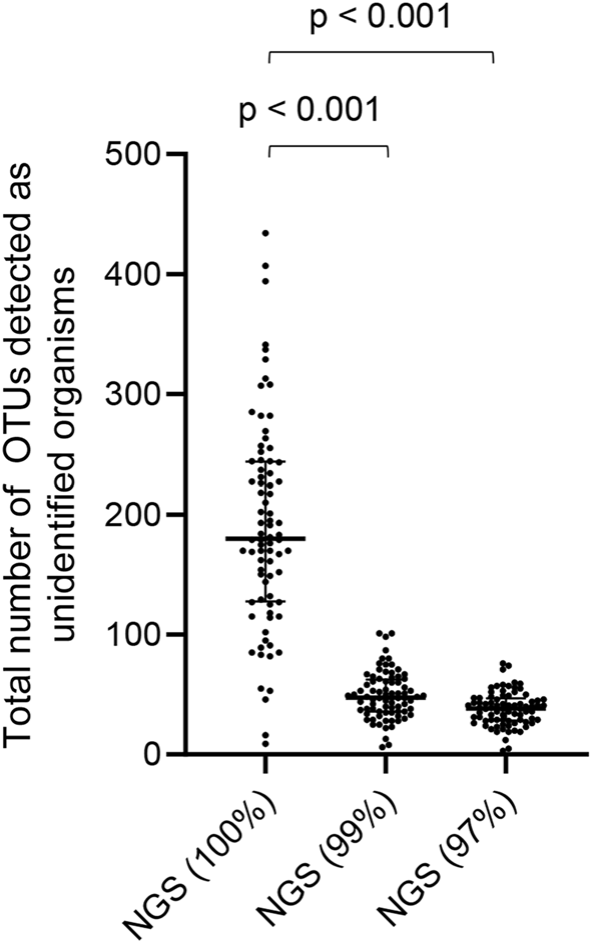
Total number of OTUs detected as unidentified organisms in each sample. The total number of unidentified organisms increased when the clustering threshold was set to 100% (*p* < 0.001). *P*-values were determined by Kruskal–Wallis test and a post hoc Dunn’s test with Bonferroni adjustment for multiple comparisons. NGS (100%) = NGS results obtained with a clustering threshold of 100%; NGS (99%) = NGS results obtained with a clustering threshold of 99%; NGS (97%) = NGS results obtained with a clustering threshold of 97%.

The total number of unidentified organisms increased when the clustering threshold was set to 100% (median 180 [IQR 128–244]) compared to 99% (48 [36–63]) and 97% (39 [27–46]) (*p* < 0.001) (Fig. 7).

## Discussion

In this study, we investigated the optimal threshold for clustering 16S rRNA gene sequences into OTUs using NGS technology (MiSeq sequencer; Illumina) by comparing the results with those obtained using the Sanger method. We demonstrated the difficulty in distinguishing the bacterial phylotype of the *S. mitis* group*,* which is the most common bacteria in respiratory infections, when the clustering threshold was set to low (< 99% OTUs). When analyzing the microbiota of respiratory infections by NGS technology, particularly in bacterial pneumonia, clustering at 100% sequence identity is necessary.

Generally, *S. pneumoniae* is the main causative agent of community-onset pneumonia^47,48^, but we previously reported that oral streptococci may also play an important role in elderly people with pneumonia with aspiration risks^30,31^. We detected *S. pseudopneumoniae* by NGS technology as the predominant phylotype in all nine cases in which *S. pneumoniae* phylotypes were detected by the Sanger method (Figs. 2 and 3). It was not possible to distinguish between *S. pneumoniae* and *S. pseudopneumoniae* because these two bacterial phylotypes were identical in the NGS amplification region (V3‒V4) used in this study (see Supplementary Fig. S2 online). Evaluating longer regions, such as the V3‒V5 region, using the MiSeq sequencer has been reported to reduce the accuracy of the second half of the read^49^. The Sanger method revealed that *S. pseudopneumoniae* dominated in one case (1.3%). Thus, the Sanger method is a better method for discriminating between these two species when examined in respiratory infections in the clinical setting.

NGS analysis with 99% and 97% homology revealed *S. pseudopneumoniae* as the predominant phylotype in 21 cases (26.3%) and 22 cases (27.5%), respectively (Figs. 2 and 3). In addition, the nine cases in which *S. oralis* was detected as the predominant phylotype by the Sanger method showed a concordance rate of 88.9% (8/9) with the results of NGS when the clustering threshold was set to 100%. However, at clustering thresholds under 100%, *S. pseudopneumoniae* was identified in all nine cases. *Streptococcus oralis* was identified as a minor population when the clustering threshold was above 99%. These findings indicate that the phylotype distinction depends on the clustering threshold; therefore, it is necessary to set the clustering threshold at 100% to estimate bacterial phylotypes more accurately.

Clustering of 16S rRNA sequences into OTUs removes minor artifactual sequence variants due to PCR amplification and sequencing errors when collapsing sequences into groups^16^. In this study, when the clustering threshold was set to 100%, the number of bacterial species defined as unidentified organisms increased. It is not possible to evaluate whether the bacterial species included in the unidentified organism group are an actual minor population or artifact. Using a high threshold, such as 100%, will result in the exclusion of samples because in several instances the number of reads falls considerably below the threshold in downstream processing; however, this can be compensated by re-sequencing the samples.

When the clustering threshold was set to 97%, some bacterial phylotypes, such as *Corynebacterium* spp. differed in the results of the Sanger method and NGS technology at the species level. Generally, the genus *Corynebacterium* is often regarded as an oral contaminant. In recent years, pneumonia due to *Corynebacterium striatum* has been reported^50,51^, and we also reported the importance of the genus *Corynebacterium* in pneumonia^29,44^, particularly when hospital-acquired^52^. The genus *Corynebacterium* has been reported to have different drug sensitivities depending on the bacterial species; therefore, species-level identification is desirable when *Corynebacterium* is suspected as the causative agent of pneumonia^53^. When the clustering threshold was set to 97%, *Corynebacterium simulans* and *C. striatum* were classified into the same OTUs. Comparison of the nucleotide sequences of the type strain (*C. simulans*, accession No. AJ012837; *C. striatum*, accession No. X84442) in the V3‒V4 amplification region for these two strains showed mismatches of three base pairs and homology of more than 99%. Therefore, when the clustering threshold was set to 97%, *C. simulans* and *C. striatum* were classified into the same OTUs by NGS technology.

There were several limitations to this study. First, this study was retrospective and included only patients with sufficient specimens available for analysis. Second, there was a difference in the analysis primer region between NGS technology and the Sanger method. Third, bacterial species with abundances of 5% or less were treated as “Others” as previously reported^29^, but there is no clear definition of the makeup of this group. In this study, we did not examine the minor population, but pathogenic bacteria, such as *Mycobacterium tuberculosis* may be included; thus, it is necessary to examine the minor population in further studies. Fourth, accurate identification of bacterial species is difficult via analysis of the 16S rRNA gene sequence alone. However, predicting the bacterial species is highly useful in deciding treatment strategies such as antibiotic selection. Therefore, it is important to utilize methods based on 16S rRNA gene sequencing that can help evaluate microbiome information as accurately as possible.

Microbiome analysis using NGS technology will continue to progress through technological innovation. When analyzing the microbiota of respiratory infections using NGS technology, it is necessary to cluster with a 100% sequence identity. Alternatively, it is necessary to use an analysis method that can maintain high accuracy for sequencing analysis, such as that for ASVs. If the analysis is performed using a low threshold on sequence clustering, important pathogens may not be identified, and/or incorrect phylotype information may be obtained.

## Supplementary Information

Supplementary Information.

## Data Availability

The sequence read data were submitted to a public database (DNA Data Bank of Japan Sequence Read Archive under the BioProject identifier PRJDB11117).
