# Supplementary material for: Refinement of microbiota analysis of specimens from patients with respiratory infections using next-generation sequencing
Source: Sci Rep. 2021 Oct 1;11:19534. doi: 10.1038/s41598-021-98985-8 (PMC8486753; doi:10.1038/s41598-021-98985-8)
Supplement: Supplementary file 1 — Supplementary Information. [file 41598_2021_98985_MOESM1_ESM.pdf]

Refinement of microbiota analysis of specimens from patients with respiratory infections using next-generation sequencing

Hiroaki Ikegami, MD [1], Shingo Noguchi, MD, PhD [1], Kazumasa Fukuda, PhD [2], Kentaro Akata, MD, PhD [1], Kei Yamasaki MD, PhD [1], Toshinori Kawanami, MD, PhD [1], Hiroshi Mukae, MD, PhD [3], Kazuhiro Yatera, MD, PhD [1,*]

[1] Department of Respiratory Medicine, University of Occupational and Environmental Health, Japan, Kitakyushu, Japan

[2] Department of Microbiology, University of Occupational and Environmental Health, Japan, Kitakyushu, Japan

[3] Department of Respiratory Medicine, Nagasaki University Graduate School of Biomedical Sciences, Nagasaki, Japan

Table of Contents

Supplementary Figure S1. Rarefaction curves of sequenced samples..3

Supplementary Figure S2. Phylogenetic tree of type strains in the genus *Streptococcus* based on the V3-V5 (a) and V3-V4 (b) regions of the 16S ribosomal RNA 4

Supplementary Table S1. Nucleotide sequence data obtained by the Sanger method and NGS technology 5

Supplementary Table S2. Predominant phylotype by the Sanger method and NGS technology 6

Reference...10

Supplementary Figure S1. Rarefaction curves of sequenced samples

a

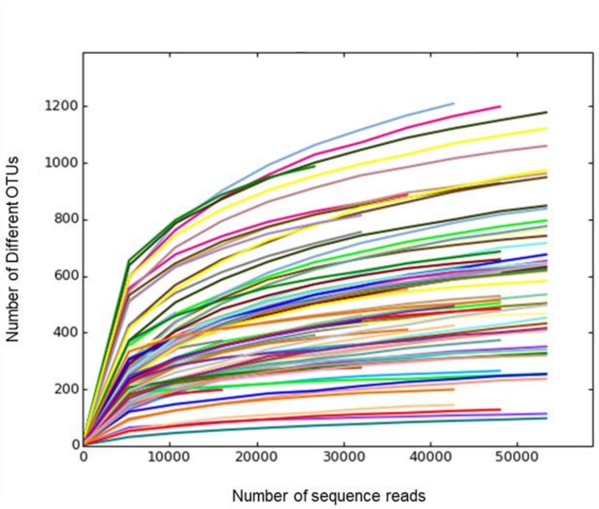

b

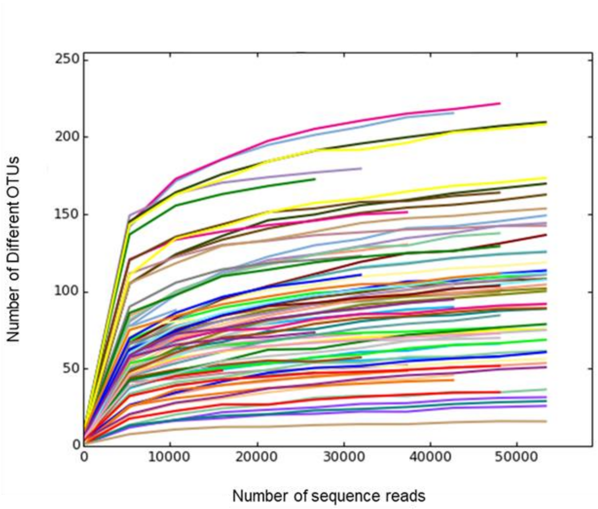

c

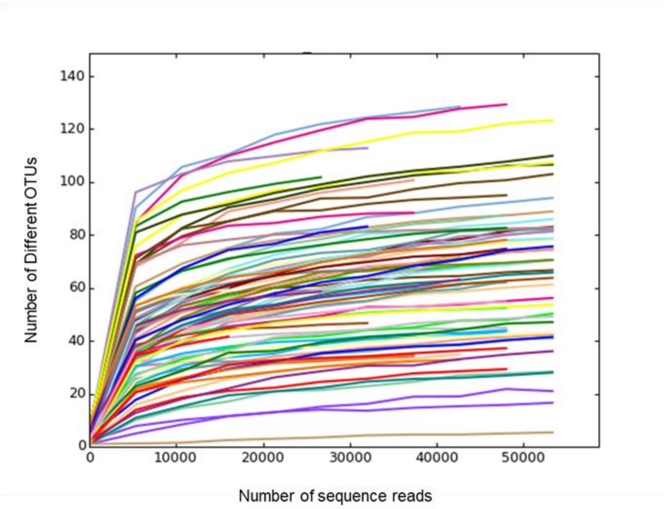

Plots were generated using Quantitative Insights into Microbial Ecology (1.8.0) [1]. The nucleotide sequences are clustered by homologies of 100% (a), 99% (b), and 97% (c). Individual colors represent each sample that was analyzed.

Supplementary Figure S2. Phylogenetic tree of type strains in the genus *Streptococcus* based on the V3-V5 (a) and V3-V4 (b) regions of the 16S ribosomal RNA

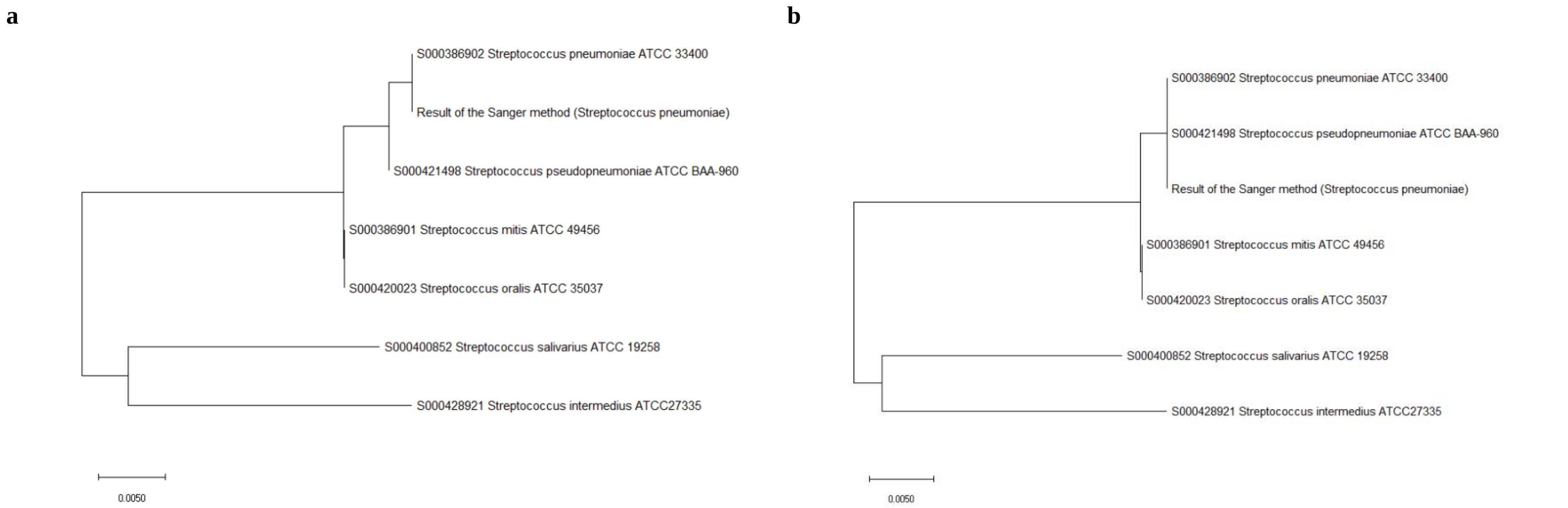

The 16S ribosomal RNA gene sequences in our case and part of *Streptococcus* type strains were aligned using MUSCLE (EMBL-EBI, Cambridge, UK) with default settings. Phylogenetic trees were constructed using the maximum likelihood method (Kimura 2-parameter model) with MEGA X software (version 10.1.8) [2].

Supplementary Table S1. Nucleotide sequence data obtained by the Sanger method and NGS technology

	clustering	Total number of clones or contigs
Sanger method		6,530
NGS technology	100%	4,103,775
	99%	4,103,775
	97%	4,103,775

Supplementary Table S2. Predominant phylotype by the Sanger method and NGS technology

a: Pneumonia with aspiration risks**, b:** Lung abscess

**a**	Sanger method	NGS technology		
Case		NGS (100%)	NGS (99%)	NGS (97%)
1	*Streptococcus pneumoniae*	*Streptococcus pseudopneumoniae*	*Streptococcus pseudopneumoniae*	*Streptococcus pseudopneumoniae*
2	*Corynebacterium simulans*	***Corynebacterium simulans***	***Corynebacterium simulans***	***Corynebacterium simulans***
3	*Streptococcus pneumoniae*	*Streptococcus pseudopneumoniae*	*Streptococcus pseudopneumoniae*	*Streptococcus pseudopneumoniae*
4	*Streptococcus pneumoniae*	*Streptococcus pseudopneumoniae*	*Streptococcus pseudopneumoniae*	*Streptococcus pseudopneumoniae*
5	*Corynebacterium striatum*	***Corynebacterium striatum***	***Corynebacterium striatum***	*Corynebacterium simulans*
6	*Streptococcus salivarius*	***Streptococcus salivarius***	***Streptococcus salivarius***	***Streptococcus salivarius***
7	*Corynebacterium simulans*	***Corynebacterium simulans***	***Corynebacterium simulans***	***Corynebacterium simulans***
8	*Streptococcus pneumoniae*	*Streptococcus pseudopneumoniae*	*Streptococcus pseudopneumoniae*	*Streptococcus pseudopneumoniae*
9	*Haemophilus influenzae*	***Haemophilus influenzae***	***Haemophilus influenzae***	***Haemophilus influenzae***
10	*Streptococcus intermedius*	***Streptococcus intermedius***	***Streptococcus intermedius***	***Streptococcus intermedius***
11	*Haemophilus influenzae*	***Haemophilus influenzae***	***Haemophilus influenzae***	***Haemophilus influenzae***
12	*Streptococcus intermedius*	***Streptococcus intermedius***	***Streptococcus intermedius***	***Streptococcus intermedius***
13	*Streptococcus oralis*	***Streptococcus oralis***	*Streptococcus pseudopneumoniae*	*Streptococcus pseudopneumoniae*
14	*Streptococcus oralis*	***Streptococcus oralis***	*Streptococcus pseudopneumoniae*	*Streptococcus pseudopneumoniae*
15	*Rothia mucilaginosa*	***Rothia mucilaginosa***	***Rothia mucilaginosa***	***Rothia mucilaginosa***
16	*Streptococcus pneumoniae*	*Streptococcus pseudopneumoniae*	*Streptococcus pseudopneumoniae*	*Streptococcus pseudopneumoniae*
17	*Fusobacterium nucleatum*	***Fusobacterium nucleatum***	***Fusobacterium nucleatum***	***Fusobacterium nucleatum***
18	*Haemophilus influenzae*	***Haemophilus influenzae***	***Haemophilus influenzae***	***Haemophilus influenzae***

19	*Moraxella catarrhalis*	***Moraxella catarrhalis***	***Moraxella catarrhalis***	***Moraxella catarrhalis***
20	*Mycoplasma pneumoniae*	***Mycoplasma pneumoniae***	***Mycoplasma pneumoniae***	***Mycoplasma pneumoniae***
21	*Streptococcus intermedius*	***Streptococcus intermedius***	***Streptococcus intermedius***	***Streptococcus intermedius***
22	*Streptococcus pseudopneumoniae*	*Streptococcus oralis*	***Streptococcus pseudopneumoniae***	***Streptococcus pseudopneumoniae***
23	*Moraxella catarrhalis*	***Moraxella catarrhalis***	***Moraxella catarrhalis***	***Moraxella catarrhalis***
24	*Escherichia coli*	***Escherichia coli***	***Escherichia coli***	***Escherichia coli***
25	*Staphylococcus aureus*	***Staphylococcus aureus***	***Staphylococcus aureus***	***Staphylococcus aureus***
26	*Haemophilus influenzae*	***Haemophilus influenzae***	***Haemophilus influenzae***	***Haemophilus influenzae***
27	*Streptococcus oralis*	***Streptococcus oralis***	*Streptococcus pseudopneumoniae*	*Streptococcus pseudopneumoniae*
28	*Neisseria perflava*	***Neisseria perflava***	***Neisseria perflava***	***Neisseria perflava***
29	*Haemophilus influenzae*	***Haemophilus influenzae***	***Haemophilus influenzae***	***Haemophilus influenzae***
30	*Haemophilus influenzae*	***Haemophilus influenzae***	***Haemophilus influenzae***	***Haemophilus influenzae***
31	*Streptococcus oralis*	***Streptococcus oralis***	*Streptococcus pseudopneumoniae*	*Streptococcus pseudopneumoniae*
32	*Streptococcus pneumoniae*	*Streptococcus pseudopneumoniae*	*Streptococcus pseudopneumoniae*	*Streptococcus pseudopneumoniae*
33	*Escherichia coli*	***Escherichia coli***	***Escherichia coli***	***Escherichia coli***
34	*Pseudomonas aeruginosa*	***Pseudomonas aeruginosa***	***Pseudomonas aeruginosa***	***Pseudomonas aeruginosa***
35	*Streptococcus salivarius*	***Streptococcus salivarius***	***Streptococcus salivarius***	***Streptococcus salivarius***
36	*Streptococcus salivarius*	*Xanthomonas vasicola*	*Xanthomonas vasicola*	*Xanthomonas vasicola*
37	*Streptococcus oralis*	***Streptococcus oralis***	*Streptococcus pseudopneumoniae*	*Streptococcus pseudopneumoniae*
38	*Streptococcus oralis*	***Streptococcus oralis***	*Streptococcus pseudopneumoniae*	*Streptococcus pseudopneumoniae*
39	*Streptococcus oralis*	***Streptococcus oralis***	*Streptococcus pseudopneumoniae*	*Streptococcus pseudopneumoniae*
40	*Streptococcus pneumoniae*	*Streptococcus pseudopneumoniae*	*Streptococcus pseudopneumoniae*	*Streptococcus pseudopneumoniae*
41	*Gemella haemolysans*	*Streptococcus oralis*	*Streptococcus pseudopneumoniae*	*Streptococcus pseudopneumoniae*
42	*Gemella haemolysans*	***Gemella haemolysans***	***Gemella haemolysans***	***Gemella haemolysans***

43	*Streptococcus macedonicus*	***Streptococcus macedonicus***	***Streptococcus macedonicus***	*Streptococcus salivarius*
44	*Prevotella veroralis*	*Veillonella atypica*	*Veillonella atypica*	*Veillonella atypica*
45	*Streptococcus pneumoniae*	*Streptococcus pseudopneumoniae*	*Streptococcus pseudopneumoniae*	*Streptococcus pseudopneumoniae*

**b**	Sanger method	NGS technology		
Case		NGS (100%)	NGS (99%)	NGS (97%)
1	*Streptococcus intermedius*	***Streptococcus intermedius***	***Streptococcus intermedius***	***Streptococcus intermedius***
2	*Prevotella melaninogenica*	***Prevotella melaninogenica***	***Prevotella melaninogenica***	***Prevotella melaninogenica***
3	*Parvimonas micra*	***Parvimonas micra***	***Parvimonas micra***	***Parvimonas micra***
4	*Klebsiella aerogenes*	***Klebsiella aerogenes***	***Klebsiella aerogenes***	*Klebsiella pneumoniae*
5	*Pseudomonas aeruginosa*	***Pseudomonas aeruginosa***	***Pseudomonas aeruginosa***	***Pseudomonas aeruginosa***
6	*Parvimonas micra*	***Parvimonas micra***	***Parvimonas micra***	***Parvimonas micra***
7	*Streptococcus intermedius*	***Streptococcus intermedius***	***Streptococcus intermedius***	***Streptococcus intermedius***
8	*Fusobacterium nucleatum*	***Fusobacterium nucleatum***	***Fusobacterium nucleatum***	***Fusobacterium nucleatum***
9	*Fusobacterium nucleatum*	***Fusobacterium nucleatum***	***Fusobacterium nucleatum***	***Fusobacterium nucleatum***
10	*Fusobacterium nucleatum*	*Veillonella atypica*	*Streptococcus pseudopneumoniae*	*Streptococcus pseudopneumoniae*
11	*Streptococcus salivarius*	***Streptococcus salivarius***	***Streptococcus salivarius***	*Streptococcus pseudopneumoniae*
12	*Eubacterium nodatum*	***Eubacterium nodatum***	***Eubacterium nodatum***	***Eubacterium nodatum***
13	*Fusobacterium nucleatum*	***Fusobacterium nucleatum***	***Fusobacterium nucleatum***	***Fusobacterium nucleatum***
14	*Streptococcus oralis*	*Rothia mucilaginosa*	*Streptococcus pseudopneumoniae*	*Streptococcus pseudopneumoniae*
15	*Haemophilus influenzae*	***Haemophilus influenzae***	***Haemophilus influenzae***	***Haemophilus influenzae***
16	*Fusobacterium nucleatum*	***Fusobacterium nucleatum***	***Fusobacterium nucleatum***	***Fusobacterium nucleatum***
17	*Porphyromonas catoniae*	***Porphyromonas catoniae***	***Porphyromonas catoniae***	***Porphyromonas catoniae***
18	*Veillonella parvula*	*Pseudomonas veronii*	*Pseudomonas veronii*	*Veillonella dispar*

19	*Klebsiella pneumoniae*	***Klebsiella pneumoniae***	***Klebsiella pneumoniae***	***Klebsiella pneumoniae***
20	*Streptococcus oralis*	***Streptococcus oralis***	*Streptococcus pseudopneumoniae*	*Streptococcus pseudopneumoniae*
21	*Fusobacterium nucleatum*	*Porphyromonas endodontalis*	***Fusobacterium nucleatum***	***Fusobacterium nucleatum***
22	*Haemophilus influenzae*	***Haemophilus influenzae***	***Haemophilus influenzae***	***Haemophilus influenzae***
23	*Streptococcus intermedius*	***Streptococcus intermedius***	***Streptococcus intermedius***	***Streptococcus intermedius***
24	*Streptococcus pneumoniae*	*Streptococcus pseudopneumoniae*	*Streptococcus pseudopneumoniae*	*Streptococcus pseudopneumoniae*
25	*Streptococcus intermedius*	***Streptococcus intermedius***	***Streptococcus intermedius***	***Streptococcus intermedius***
26	*Streptococcus intermedius*	***Streptococcus intermedius***	***Streptococcus intermedius***	***Streptococcus intermedius***
27	*Prevotella oris*	***Prevotella oris***	***Prevotella oris***	***Prevotella oris***
28	*Staphylococcus aureus*	***Staphylococcus aureus***	***Staphylococcus aureus***	***Staphylococcus aureus***
29	*Staphylococcus aureus*	***Staphylococcus aureus***	***Staphylococcus aureus***	***Staphylococcus aureus***
30	*Klebsiella variicola*	***Klebsiella variicola***	***Klebsiella variicola***	*Klebsiella pneumoniae*
31	*Streptococcus salivarius*	***Streptococcus salivarius***	***Streptococcus salivarius***	***Streptococcus salivarius***
32	*Streptococcus intermedius*	***Streptococcus intermedius***	***Streptococcus intermedius***	***Streptococcus intermedius***
33	*Streptococcus salivarius*	***Streptococcus salivarius***	***Streptococcus salivarius***	***Streptococcus salivarius***
34	*Streptococcus intermedius*	***Streptococcus intermedius***	***Streptococcus intermedius***	***Streptococcus intermedius***
35	*Streptococcus intermedius*	***Streptococcus intermedius***	***Streptococcus intermedius***	***Streptococcus intermedius***

For results that were the same between NGS technology and the Sanger method, the strain name is presented in bold font.

Definition of abbreviations: NGS (100%) = NGS results obtained with a clustering threshold of 100%; NGS (99%) = NGS results obtained with a clustering threshold of 99%; NGS (97%) = NGS results obtained with a clustering threshold of 97%.

Reference

1. Caporaso, J. G. *et al.* QIIME allows analysis of high-throughput community sequencing data. *Nat Methods*. **7**, 335-336 (2010).

2. Kumar, S., Stecher, G., Li, M., Knyaz, C. & Tamura, K. MEGA X: Molecular Evolutionary Genetics Analysis across Computing Platforms. *Mol Biol Evol*. **35**, 1547-1549 (2018).
